# Development and Pretesting of Hookah Tobacco Public Education Messages for Young Adults

**DOI:** 10.3390/ijerph17238752

**Published:** 2020-11-25

**Authors:** Lilianna Phan, Andrea C. Villanti, Glenn Leshner, Theodore L. Wagener, Elise M. Stevens, Andrea C. Johnson, Darren Mays

**Affiliations:** 1 Cancer Prevention & Control Program, Lombardi Comprehensive Cancer Center, Georgetown University Medical Center, 3300 Whitehaven Street NW, Suite 4100, Washington, DC 20007, USA; lilianna.phan@nih.gov; 2 Vermont Center on Behavior and Health, Department of Psychiatry, University of Vermont, 1 South Prospect Street MS 482, Burlington, VT 05401, USA; andrea.villanti@uvm.edu; 3 Gaylord College of Journalism and Mass Communication, University of Oklahoma, 3520B Gaylord Hall, Norman, OK 73019, USA; leshnerg@ou.edu; 4 Center for Tobacco Research, The Ohio State University James Comprehensive Cancer Center, Department of Internal Medicine, The Ohio State University, 3650 Olentangy River Road, Suite 410, Columbus, OH 43214, USA; theodore.wagener@osumc.edu; 5 Harvard T.H. Chan School of Public Health, Harvard University, 677 Huntington Ave, Boston, MA 02115, USA; estevens@hsph.harvard.edu; 6 Department of Psychiatry and Tobacco Center of Regulatory Science, Perlman School of Medicine, University of Pennsylvania, 3535 Market Street, Philadelphia, PA 19104, USA; Andrea.Johnson1@Pennmedicine.upenn.edu

**Keywords:** tobacco, hookah, waterpipe, public education, young adults

## Abstract

Young adults’ hookah tobacco use is fueled by misperceptions about risks, appealing flavors, and social use. We developed and pretested public education messages to prevent and reduce hookah tobacco smoking among young adults. We used a two (user status: current hookah user, susceptible never user) by two (risk content: health harms or addiction) by three (message theme: harms/addiction risk alone, harms/addiction risk flavors, or harms/addiction risk social use) design with two messages/condition (*n* = 12 total messages). Young adults aged 18–30 (*N* = 713) were randomized to 1 of 12 messages and completed measures assessing message receptivity, attitudes, and negative emotional response. Harms messages were associated with greater receptivity (*p* < 0.001), positive attitudes (*p* < 0.001), and negative emotional response (*p* < 0.001) than addiction messages. Messages with harm or addiction content alone were associated with greater receptivity than social use-themed messages (*p* = 0.058). Flavor-themed messages did not differ in receptivity from harm or addiction content alone or social use-themed messages. Messages about the health harms of hookah tobacco use resonate more with young adults than addiction risk messages. Social use-themed messages produce the lowest receptivity. These findings can guide population-based approaches to communicate hookah tobacco risks to young adults.

## 1. Introduction

U.S. young adults are increasingly initiating hookah tobacco use and many young adults are current hookah users [1]. Recent U.S. population data indicate 9.2% of young adults are current (i.e., past 30 day) hookah smokers, and young adults had the highest prevalence of current hookah use from 2013 to 2016 [1]. One hookah tobacco smoking session lasts approximately an hour or more and exposes users to higher levels of a variety of harmful toxicants consistent with those found in cigarettes, including nicotine, carbon monoxide, heavy metals, and aldehydes [2,3,4,5]. Evidence indicates health harms are associated with even infrequent exposures to hookah tobacco smoking [2,6] and long-term hookah tobacco use increases risks for cancer, pulmonary and cardiovascular disease, and other negative health outcomes [5,7,8]. Additionally, hookah tobacco use has been linked to nicotine dependence [9,10] and subsequent cigarette smoking [11,12,13] among young adults during a critical developmental period vulnerable to the acceleration of tobacco use [14].

Despite evidence indicating the risks of health harms and addictiveness of hookah tobacco smoking, young adults’ interest and use of hookah tobacco are associated with misperceptions about these risks, heavily marketed flavorings that appeal to young adults, and social aspects of use [15,16,17,18,19,20,21]. Data from the 2013–2014 Population Assessment of Tobacco and Health (PATH) Study indicate that among U.S. adult hookah tobacco users ≥18 years, some are daily and weekly users (11%) but monthly (14%), less than monthly (42%), and annual use (34%) are more common [22]. Both young adult hookah users and non-users perceive hookah tobacco use to be safer than cigarette smoking and health harm risks to be less serious or likely to happen [16]. Infrequent patterns of use also fuel young adults’ self-characterization as social hookah users [23], lending to false perceptions that they are not at risk of nicotine addiction and can quit hookah tobacco easily [15]. Evidence suggests that hookah tobacco users experience withdrawal symptoms, alter behavior to access hookah tobacco, and have a hard time quitting despite wanting to quit [9]. Additionally, although flavorings like fruit and candy present substantial risks of health harm and addiction [24,25], flavored tobacco products including hookah tobacco are perceived to be more appealing, better tasting, and less dangerous than non-flavored products [26]. A longitudinal analysis using PATH data demonstrated the role of flavors in initiation and use: approximately 66% of young adult hookah users reported that their first use was flavored, and initiation with flavored hookah tobacco was prospectively associated with current use [27]. Similarly, a recent study found that 80% of young adult hookah users used flavored hookah tobacco in the past 30 days [28].

Despite this evidence on the factors driving hookah use in young adults, there is limited research on how to communicate about the risks of hookah tobacco use to this population. Prior studies testing brief hookah tobacco health harms and addiction risk messaging among young adults found promising evidence that targeting these risk beliefs may help curb hookah use. Overall, these studies found exposure to such messaging increased perceived risk and worry about health harm and addiction [29,30,31], decreased willingness and curiosity to try hookah tobacco among susceptible non-users [30], and increased cessation among users [29]. Mays and colleagues compared the effects of messages about health harms and the potential added effects of combining health harms and addiction message content [31]. They found that adding addiction content to content about health harms did not produce stronger effects on risk perceptions or motivation to quit in comparison with those focusing on health harms only and suggested that their addiction messaging may have focused on the addiction potential of daily use, and may not have resonated with young adult social hookah users. Given the non-daily social nature of hookah use, it may be important to directly target these use patterns in risk communication messaging. Similarly, it may be important to address other key drivers of hookah use such as product flavors in such messaging for young people. Research on how to optimally design messaging about the risks of hookah tobacco can inform population-based efforts to communicate these risks to young adult consumers. For example, the Food and Drug Administration (FDA) engages in public education messaging on cigarettes [32,33], electronic cigarettes, and smokeless tobacco [34] and is positioned to engage in similar public education on hookah tobacco use [35,36]. Evidence on effective public education messaging for hookah is crucial to inform such efforts by the FDA and other public health agencies. Brief public education messaging such as those developed in this study could be implemented in various settings popular to young adults, including social media or other online platforms [33,37].

This study builds from prior work on hookah tobacco messaging by systematically developing and pretesting public education messages that target the health harms and addictiveness of hookah tobacco, as well as two factors associated with initiation and continued use: flavors and social use. To date, risk communication research has not examined the impact of coupling messaging about health harms and addiction risks with flavors and social use. Understanding how young adult hookah tobacco consumers react to such messaging will provide insights on optimal public education messaging that could in turn influence intentions to use hookah tobacco, and ultimately change use behavior among current users [38]. The objective of this study was to develop and test message content designed to prevent and reduce hookah tobacco use among young adult current hookah users and susceptible never users.

## 2. Materials and Methods

### 2.1. Setting

Similar to prior studies testing tobacco message effects [30,31], we recruited study participants in September 2018 using convenience sampling through the crowdsourcing website Amazon Mechanical Turk (MTurk) for an online experiment. Although this online recruitment generates a convenience sample, recent evidence demonstrates correlational and experimental studies conducted through MTurk for tobacco research produce comparable effects to those using population-based samples [39,40]. We used several data quality assurance measures, including prohibiting duplicate responses, manually reviewing completed screeners to identify potential duplicate or fraudulent responses, requiring verification to prevent automated completion (i.e., by bots), and using randomly generated completion codes [41,42].

### 2.2. Participants

Eligible participants were young adults aged 18–30 years who either (1) self-reported smoking hookah tobacco at least once in the past month or (2) had never smoked hookah tobacco but were deemed to be susceptible based on a valid measure [30]. Participation was also restricted to those with MTurk accounts registered in the U.S. Our goal was to recruit at least 50 current hookah users and 50 susceptible never users in each of the six experimental conditions (i.e., 600 or more total participants) to achieve adequate statistical power to detect mean differences in outcomes comparable to similar, previous studies [30,31].

### 2.3. Study Design

All study procedures occurred online at a single timepoint. Potential participants reviewed a brief study description then proceeded to questions to determine their eligibility. Eligible participants reviewed a complete study description and an online informed consent form. After providing informed consent, participants completed initial measures of demographic characteristics, hookah tobacco use (current hookah users only), intentions to smoke hookah (susceptible never users only), cigarette smoking, and other tobacco product use. Prior to questions about hookah tobacco use, instructions indicated “the following ask about smoking tobacco in a hookah. Hookah tobacco is also called other names including waterpipe, shisha, and narghile” and included an image of a hookah above this description. Participants were then randomized to view hookah tobacco public education messages. The experiment was a 2 (user status: current hookah user, susceptible never user) by 2 (risk content: health harms or addiction) by 3 (message theme: harms/addiction risk alone, harms/addiction risk, flavors, or harms/addiction risk, social use) design. Participants viewed one message in the condition to which they were randomized for as long as they wished, then proceeded to complete post-exposure measures of study outcomes. Participants were compensated $2.00. All participants provided informed consent before they participated, the host institution’s institutional review board reviewed and approved all study procedures (Protocol # 2018-0390), and all study procedures were carried out following the Declaration of Helsinki.

### 2.4. Message Content

We developed text-based message content using an iterative process where we created draft messages, received rounds of feedback from an interdisciplinary group of experts on our research team, and revised the content following each round of review. We repeated this process until there was consensus on the messages to be tested.

To develop message content, we drew from research on the risks of health harms and addictiveness of hookah tobacco [6,43,44] and prior studies testing the effects of messages communicating these risks [30,31,45]. We also targeted message content at young adults’ beliefs about the risks of hookah tobacco use that are associated with use behavior [46,47,48,49]. Finally, we developed content for message themes focused on flavors and social use based on evidence of the wide array of hookah tobacco flavors that are commonly used by young people and research indicating young adults’ hookah tobacco use is driven by beliefs that social use poses little risk [15,16,17,18,19,20,21,46,50,51,52,53]. This content was additionally based on evidence of the potential harmful exposures from smoking flavored hookah tobacco and from smoking hookah tobacco socially [6,24].

Based on this evidence, we developed message content for themes within a 2 (risk content: health harms or addiction) by 3 (message theme: harms/addiction risk alone, harms/addiction risk, flavors, or harms/addiction risk, social use) design. The 6 experimental conditions were: harms risk alone; harms risk, flavors; harms risk, social use; addiction risk alone; addiction risk, flavors; and addiction risk, social use. We developed message text that was similar in structure, content, and length and developed a common layout to ensure similar visual presentation across all of the messages. Each message consisted of evidence-based risk information in lay language specific to each condition. On average, each message contained 22 words and one hashtag, #UnfollowHookah, which was included to align message content with social platforms to which young adults are commonly exposed to such communication. Once text for each message was finalized, we randomly assigned 1 of 4 images relevant to hookah tobacco use: a hookah, social hookah smoking setting, a smoke cloud, and a ring of smoke to pair with each message text. The product of our message development process was 12 total messages, or 2 messages in each of the 6 experimental conditions (Supplementary Figure S1).

### 2.5. Measures

#### 2.5.1. Demographic Characteristics

Demographic characteristics assessed included sex, age, race, ethnicity, educational attainment, employment status, and subjective financial situation [54,55].

#### 2.5.2. Susceptibility to Using Hookah Tobacco

At eligibility screening, among those who had never smoked hookah tobacco, we assessed susceptibility to smoking hookah using a 4-item measure from prior research [30]. The four items were as follows: Do you think that you will smoke tobacco from a hookah soon? Do you think that you will smoke tobacco from a hookah in the next year? Do you think that in the future you might experiment with hookah tobacco smoking? If one of your best friends asked you to smoke tobacco from a hookah, would you? The response options were as follows: Definitely yes; Probably yes; Probably no; Definitely no. Participants were considered susceptible if they gave a response other than Definitely no to any item. Participants responding Definitely no to all items were considered to be non-susceptible and were not eligible to participate in the study.

Among susceptible never users, we also captured their willingness to smoke hookah in the future using 4 items adapted from prior studies [56,57]. The items captured how likely participants would be to smoke hookah tobacco in the future if offered it by a friend, how tempted they are to smoke hookah tobacco in the next year, if they saw themselves smoking hookah tobacco in the next year, and how curious they were about smoking hookah tobacco. Responses to all items were on 1 (Not at all) to 7 (Very) scale, and we averaged responses to create a score where higher values indicate stronger willingness to smoke hookah (Cronbach’s α = 0.88, McDonald’s Ω = 0.91).

#### 2.5.3. Hookah Tobacco Use

We assessed hookah tobacco use at eligibility screening by first asking participants, “Have you ever smoked tobacco from a hookah, even 1 or 2 puffs?” Among those answering yes, we then asked on how many of the past 30 days they had smoked hookah tobacco. Among current hookah users, we also assessed frequency of hookah smoking (daily/weekly or monthly), and their motivation to quit on a 1 (Not at all) to 7 (Very) scale [31]. Participants who did not smoke hookah tobacco in the past 30 days were not eligible to participate in the study.

#### 2.5.4. Other Tobacco Use

We assessed cigarette smoking with two items to define current smokers as those who had smoked 100 or more lifetime cigarettes and currently smoked cigarettes every day or some days [54]. For descriptive purposes, we report the proportion of current cigarette smokers and nonsmokers. We also measured if participants used electronic cigarettes, large cigars, little cigars/cigarillos, and/or smokeless tobacco in the past 30 days. For descriptive purposes, we report the proportion of those individuals using any other tobacco product in the past 30 days (i.e., current tobacco user) versus not (i.e., non-tobacco user).

#### 2.5.5. Message Manipulation Checks

To confirm the message themes in the 2 × 3 design worked as intended, we created manipulation check items similar to prior work [58]. After the message exposure, participants responded to the following: 1. The message focused on the health risks of smoking hookah tobacco; 2. The message focused on the addictiveness of smoking hookah tobacco; 3. The message focused on the flavors in hookah tobacco; 4. The message focused on social aspects of smoking hookah tobacco, such as sharing with friends or smoking at hookah lounges or cafes. Responses ranged from 1 (Strongly disagree) to 7 (Strongly agree). We analyzed these items individually.

#### 2.5.6. Message Receptivity

We assessed message receptivity with 9 items that capture participants’ agreement with statements about the messages on a 1 (Strongly disagree) to 7 (Strongly agree) scale [45,59,60]. Example items include the following: The message grasped my attention; The message was convincing; The message gave me good reasons why I should not smoke hookah tobacco; Overall, the message was effective. We averaged responses to the 9 items to create a score, with a range of 1 to 7 and higher values indicating greater message receptivity (Cronbach’s α = 0.92, McDonald’s Ω = 0.92).

#### 2.5.7. Message Attitudes

We measured attitudes toward the messages using 9 items adapted from prior research [30]. Items assessed participants’ attitudes towards the content of the messages using bipolar responses with word pairs appearing at the ends of the −3 to 3 scale. Examples of attitudes assessed included boring/exciting, not stimulating/stimulating, not engaging/engaging, common/unique, and weak visuals/strong visuals. We averaged responses to the items to create a score, with a range of 1 to 7 and higher values indicating more positive attitudes (Cronbach’s α = 0.94, McDonald’s Ω = 0.94).

#### 2.5.8. Negative Emotional Response

We measured negative emotional response to the messages, a response that is critical to the efficacy of tobacco messages, with 4 items capturing the extent to which participants felt frightened, anxious, nervous, and worried in response to the message [61,62]. Responses ranged from 1 (Not at all) to 4 (Extremely), and we averaged item responses to create a score, with a range of 1 to 4 and higher values indicating stronger negative emotional response (Cronbach’s α = 0.91, McDonald’s Ω = 0.91).

#### 2.5.9. Statistical Analyses

Our statistical analyses included several steps. First, we characterized the sample using descriptive statistics. Next, for the manipulation check items we used one-way analysis of variance (ANOVA) with a six-level independent variable reflecting messages tested in the 2 (risk content) by 3 (message theme) design. We examined the main effect for this six-level variable and post hoc pair-wise comparisons of means for manipulation check items adjusting for hookah user status as a covariate. For message receptivity, attitudes, and negative emotional response, we used a 2 (user status) by 2 (risk content) by 3 (message theme) ANOVA with the main effect for each factor and interactions between each factor included. We included a variable for image displayed within each condition as a covariate to account for this aspect of our design in the analysis. We examined statistical significance of all main and interaction effects and post hoc pair-wise comparisons of means for statistically significant main and interaction effects. We used Bonferroni adjusted *p*-values for post hoc pair-wise comparisons of means to account for multiple statistical tests.

## 3. Results

### 3.1. Sample Characteristics

Overall, 2583 individuals completed eligibility screening. Of these, 1852 (71.7%) were ineligible and 731 (28.3%) met eligibility criteria. Of those eligible, 333 susceptible never users and 380 current users (*N* = 713; 97.5% of eligible participants) completed study procedures. Table 1 displays characteristics of the sample.

### 3.2. Message Manipulation Checks

Table 2 displays least square mean differences from the ANOVAs examining manipulation check items to confirm message content and themes we developed operated as intended. Participants indicated on average that messages with harms risk content focused on health harms of hookah smoking significantly more than those with addiction risk content (Table 2). Participants indicated messages with addiction risk content focused on the addictiveness of hookah smoking significantly more than those with harms risk content. Participants indicated that messages with flavors and social use themes focused significantly more on flavors in hookah tobacco and social smoking, respectively, compared with those without these themes (Table 2). These results support the success of our message risk content and theme manipulations.

### 3.3. Message Receptivity

For message receptivity, there was a statistically significant main effect for risk content (*F*_1,685_ = 23.4, *p* < 0.001) and the main effect for message theme approached significance (*F*_2,685_ = 5.3, *p =* 0.056; Table 3). Messages with harms risk content produced significantly greater message receptivity (*M* 5.1, *SE* 0.07) than those with addiction risk content (*M* 4.6, *SE* 0.07; Table 4). Messages with harms or addiction risk content alone (*M* 5.1, *SE* 0.07) produced greater receptivity than those with harms or addiction risk content themed as social use (*M* 4.6, *SE* 0.07, *p =* 0.058; Table 4). The interaction between risk content and message theme also approached significance (*F_2,285_ =* 2.9, *p =* 0.056). Pair-wise comparisons for this interaction effect indicated addiction messages themed as social use (*M* 4.3, *SE* 0.12) produced significantly lower receptivity than harms messages themed as social use (*M* 5.1, *SE* 0.13, *p* < 0.001). Similarly, addiction messages themed as flavor (*M* 4.6, *SE* 0.12) produced significantly lower receptivity than harms messages themed as flavors (*M* 5.0, *SE* 0.13, *p =* 0.031).

### 3.4. Message Attitudes

For message attitudes, the only statistically significant effect was the main effect for risk content (*F*_1,696_ = 10.4, *p* < 0.001; Table 3). Messages with harms risk content generated significantly more positive message attitudes (*M* 4.8, *SE* 0.08) than those with addiction risk content (*M* 4.4, *SE* 0.08; Table 4).

### 3.5. Negative Emotional Response

For negative emotional response, there were statistically significant main effects for hookah user status (*F*_1,698_ = 16.2, *p* < 0.001) and risk content (*F*_1,698_ = 15.4, *p* < 0.001; Table 3). Susceptible never users had significantly lower negative emotional response to the messages (*M* 1.8, *SE* 0.05) than current hookah users (*M* 2.1, *SE* 0.05; Table 4). Messages with harms risk content generated significantly greater negative emotional response (*M* 2.1, *SE* 0.05) than messages with addiction risk content (*M* 1.8, *SE* 0.05; Table 4).

## 4. Discussion

This study developed and pretested public education messages communicating the risks of hookah tobacco use among young adult current hookah users and susceptible never users. We developed messages communicating risks of health harms and addictiveness of hookah tobacco smoking, and coupled these risks with message themes targeted to the appeal of hookah tobacco flavors and social hookah tobacco use. This study uniquely tested novel public education message content designed for delivery through media that young adults frequently use (e.g., social media) and targeted the patterns and motives of young adults’ hookah tobacco use. Our manipulation check results indicate the message content we created successfully communicated the intended risks of health harms and addictiveness and themes about flavoring and social use. Overall, our findings indicate messages communicating about the risk of health harms from hookah tobacco use performed better on the outcomes assessed than those communicating about addiction risks. This is consistent with prior studies testing risk-based messaging for hookah tobacco use among young adults [30,31]. Our study also found that young adult hookah tobacco users and susceptible never users were less receptive to messages targeting social use compared with those addressing health harm or addiction risks alone. Messages addressing flavors performed similarly on all outcomes compared with those addressing social use or addressing only health harm or addiction risks. Importantly, constructs similar to our measures, including message receptivity, have been demonstrated in prior research to correlate with stronger tobacco-related beliefs and behavioral outcomes after message exposure [63,64]. This supports the value and potential impact of using these message-oriented outcomes to identify optimal message content.

This study adds to research on tobacco communication in several ways. This is one of the first studies to provide evidence of hookah tobacco message effects on outcomes that are message-oriented, including message receptivity, attitudes about the messages, and negative emotional response. Prior studies have primarily tested message content that is longer and requires more energy and effort for participants to review and process than messages examined in this study [31,56]. Our message content targeted new themes associated with young adult interest and use of hookah tobacco and was presented to participants as a single, brief message designed for implementation in contexts such as public education campaigns or other online media platforms (e.g., social media).

The finding that messages communicating about addiction risks did not perform as well as those communicating about the risks of health harms is consistent with the published literature [31,65]. There is evidence indicating that young adult hookah users do not think of hookah tobacco as being addictive. Rather, they believe that social use does not lead to addiction and that they will quit before they become addicted [65]. Young adults also may not identify symptoms of nicotine withdrawal (e.g., sleep disturbance [66]), which could contribute to their beliefs about low addiction risk. Though our messages successfully targeted addiction risks, as indicated by manipulation check results, it is possible the addiction risk content in these messages was not sufficiently strong or did not convey relatable aspects of nicotine addiction to this population. We also did not test whether messages combining health harm and addiction risk content increase the effects on measured outcomes, however one previous study demonstrated hookah tobacco messages combining health harm and addiction risk content did not produce stronger effects compared with messages communicating only risks of health harms [31].

Our findings, taken together with prior studies [31,65], highlight the challenges of communicating the risks of addiction from hookah tobacco smoking in a way that resonates with young adults. Future research is needed to examine the strength of addiction risk claims and other strategies for communicating addiction risks that may resonate more strongly with young adults. For example, for recent public education messaging about the risks of cigarette smoking aimed at youth, research indicates that specific message content about addiction vetted with the target audience, such as loss of control and financial costs of smoking, was successful [67]. Similar strategies will be useful to identify optimal ways to communicate about the addiction risks of hookah tobacco to young adults. Other recent studies highlight the impact of perceived source credibility in tobacco-related messaging [68,69]. We did not assess source credibility in this study, but it is a message feature that is important to examine in relation to addiction risk messaging in future studies.

This study is among the first to design and test message content specifically communicating to young adults about the risks of flavored hookah tobacco and social hookah tobacco smoking. As noted above, our work uniquely adds to the hookah tobacco risk communication research [30,31,70], expanding beyond messages about risks of health harms and/or addiction with content targeting known factors that make hookah tobacco appealing to young adults. Overall, we found that messages themed about flavored hookah tobacco performed similar to others tested, such as those about the risks of health harms or addiction alone. However, participants reported lower receptivity to messages about social use compared with those communicating about health harms or addiction risks alone. Social use is the most prevalent pattern of hookah tobacco smoking among young adults in the U.S. [22], thus they may be less receptive to messages about social hookah smoking because it is a behavior they engage in (i.e., hookah users) or that they are at risk of in the future and may observe among their peers (i.e., susceptible never users). In future studies, measures of counterarguing or defensive processing of messages [71,72,73] will be useful to capture to better understand how young adults respond to such messages.

Strengths of our study include the experimental design and our iterative approach to message content development, though the results should be interpreted in light of study limitations. We relied on an online convenience sample, which reduces generalizability to other groups of young adults. There is, however, evidence that tobacco message testing and other experimental research conducted with online convenience samples produces similar findings to studies conducted in population-based samples, somewhat mitigating against this concern [39,40]. We implemented several steps to ensure data quality due to the crowdsourced nature of data collection, however data quality remains a paramount concern in crowdsourced studies [74]. Additionally, though our findings support that communication about the risks of health harms from hookah tobacco use may be optimal in public education messaging, our experimental design does not allow us to identify specific risks (e.g., cancer, infection) within this theme that may be more or less effective. We compared messages against one another, not to a control condition, because our goal was to draw comparisons across messages with different content. Consequently, our design does not provide information about how responses to the messages compare to an unexposed group or to a group exposed to message content unrelated to hookah tobacco. Finally, the cross-sectional experimental design does not provide information about changes in outcomes over time. This will be important to address in future studies.

## 5. Conclusions

Our study findings favor messages communicating about health harm risks over those conveying addiction risks, and suggest messages communicating the risks of flavored hookah tobacco smoking perform relatively well among young adults. Messages about addiction risks did not perform as well, particularly content about the risk of addiction from social hookah tobacco use. Research is needed to continue to improve message content conveying the addictiveness of hookah tobacco use to young adults. From a practical perspective, these findings can inform population-based efforts to communicate with vulnerable groups of young people about the risks of hookah tobacco use, such as public education campaigns. They also raise important issues to be addressed in future work to develop and study the effects of hookah tobacco public education messages among young adults.

## Figures and Tables

**Table 1:** Sample characteristics.

Demographics	Mean (SD)	*n* (%)
Sex		
Male		479 (67.2)
Female		220 (30.9)
Age	26.8 (2.7)	
Ethnicity		
Non-Hispanic		556 (78.0)
Hispanic		139 (19.5)
Race		
Non-White		198 (27.8)
White		495 (69.4)
Education		
High school education/GED or less		86 (12.1)
Some college education		269 (37.7)
College education or higher		343 (48.0)
Employment		
Not full-time employed		170 (23.8)
Full-time employed		521 (73.1)
Subjective Financial Status		
Meets basic expenses or less		206 (28.9)
Higher than basic expenses		492 (69.0)
Hookah user status		
Susceptible never user		333 (46.7)
Current hookah user		380 (53.3)
Hookah willingness to smoke ^🟄^	3.6 (1.6)	
Hookah use frequency ^■^		
Daily/Weekly		202 (53.3)
Monthly		177 (46.7)
Motivation to quit ^■^	4.0 (2.1)	
Cigarette smoking status		
Nonsmoker		360 (50.5)
Current smoker		339 (47.5)
Other tobacco use		
Non-tobacco user		577 (80.9)
Current tobacco user		123 (17.3)

Note: Some *n* totals for categories within variables do not sum to total sample size due to sporadic missing data (<5% of cases for any individual variable). ^🟄^ indicates susceptible never users; ^■^ indicates current hookah users.

**Table 2:** Least squares mean differences for manipulation check items.

	The Message Focused on…			
Risk Content and Message Theme	Health Harms (*M, SE*)	Addictiveness (*M, SE*)	Flavors (*M, SE*)	Social Smoking (*M, SE*)
Harms Risk Alone (A)	6.0 (0.15) ^D,E,F^	3.4 (0.17) ^D,E,F^	2.5 (0.18) ^B,E^	3.0 (0.18) ^C,E,F^
Harms Risk, Flavors (B)	5.6 (0.16) ^D,E,F^	3.9 (0.18) ^D,E,F^	5.0 (0.19) ^A,C,D,E,F^	3.5 (0.19) ^C,F^
Harms Risk, Social Use (C)	5.7 (0.16) ^D,E,F^	3.4 (0.17) ^D,E,F^	2.9 (0.19) ^B,E^	5.3 (0.18) ^A,B,D,E,F^
Addiction Risk Alone (D)	4.8 (0.15) ^A,B,C, E, F^	6.2 (0.16) ^A,B,C,E,F^	2.8 (0.17) ^B,E^	3.2 (0.17) ^C,F^
Addiction Risk, Flavors (E)	3.8 (0.16) ^A,B,C, D^	5.3 (0.17) ^A,B,C,D^	5.8 (0.19) ^A,B,C,D,F^	3.5 (0.18) ^A,C,F^
Addiction Risk, Social Use (F)	3.7 (0.16) ^A,B,C, D^	5.2 (0.17) ^A,B,C,D^	2.7 (0.18) ^B,E^	5.8 (0.18) ^A,B,C,D,E^

Note: Means with different superscript letters within a column differ significantly at *p* < 0.05 in pair-wise comparisons. Hookah tobacco user status (current user, susceptible never user) was a covariate.

**Table 3:** Analysis of variance results for message outcomes.

	Message Receptivity			Message Attitudes			Negative Emotional Response		
	*F _df_*	Partial *η*^2^	*p*	*F _df_*	Partial *η*^2^	*p*	*F _df_*	Partial *η*^2^	*p*
**Main Effects**									
User Status	1.3 _1,685_	0.002	0.249	0.301 _1,696_	0.000	0.584	**16.2 _1,698_**	**0.023**	**<0.001**
Risk Content	**23.1 _1,685_**	**0.033**	**<0.001**	**10.4**_1,696_	**0.015**	**<0.001**	**15.4 _1,698_**	**0.022**	**<0.001**
Message Theme	2.9 _2,685_	0.008	0.056	0.035 _2,696_	0.000	0.966	1.32 _2,698_	0.004	0.269
**Interaction Effects**									
User Status x Risk Content	0.284 _1,685_	0.001	0.594	0.132 _1,696_	0.000	0.717	0.217 _1,698_	0.000	0.641
User Status x Message Theme	0.535 _2,685_	0.002	0.586	0.863 _2,696_	0.003	0.422	1.18 _2,698_	0.003	0.308
Risk Content x Message Theme	2.91 _2,685_	0.008	0.055	1.0 _2,696_	0.003	0.367	0.919 _2,698_	0.003	0.400

Note: *F* statistics, partial *η*^2^, *p*-values from analysis of variance. Statistically significant main and interaction effects are highlighted in bold font. Image displayed for each message within each condition was a covariate.

**Table 4:** Least squares mean differences for main effects for message outcomes.

	Message Receptivity (*M, SE*)	Message Attitudes (*M, SE*)	Negative Emotional Response (*M, SE*)
Main Effects			
User Status			
Current Hookah User (A)	4.8 (0.07)	4.7 (0.08)	2.1 (0.05) ^B^
Susceptible Never User (B)	4.9 (0.07)	4.6 (0.08)	1.8 (0.05) ^A^
Risk Content			
Harms (C)	5.1 (0.07) ^D^	4.8 (0.08) ^D^	2.1 (0.05) ^D^
Addiction (D)	4.6 (0.07) ^C^	4.4 (0.08) ^C^	1.8 (0.05) ^C^
Message Theme			
Harms/Addiction Risk Alone (E)	5.0 (0.09)	4.6 (0.09)	2.0 (0.06)
Harms/Addiction Risk, Flavors (F)	4.7 (0.09)	4.6 (0.10)	2.0 (0.06)
Harms/Addiction Risk, Social Use (G)	4.7 (0.09)	4.6 (0.10)	1.9 (0.06)

Note: Means with different superscript letters within a column differ significantly at *p* < 0.05 in pair-wise comparisons after Bonferroni adjustment to account for multiple comparisons. Image displayed for each message within each condition was a covariate.

## Supplementary Materials

The following are available online at https://www.mdpi.com/1660-4601/17/23/8752/s1, Figure S1: Message Exposures by Risk Content and Message Theme.
