# Supplementary material for: Development and Pretesting of Hookah Tobacco Public Education Messages for Young Adults
Source: Int J Environ Res Public Health. 2020 Nov 25;17(23):8752. doi: 10.3390/ijerph17238752 (PMC7728075; doi:10.3390/ijerph17238752)

Figure S1

Message Exposures by Risk Content and Message Theme

Message Theme

Risk Content — Alone | Flavors | Social Use

Harms

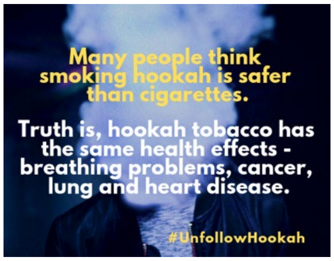

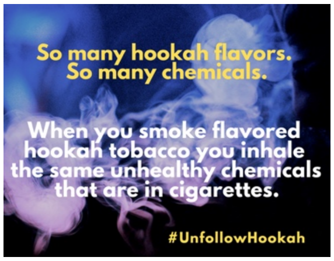

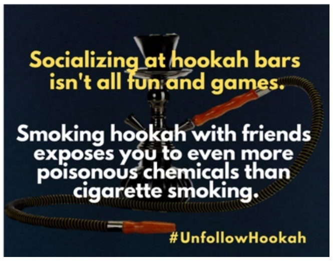

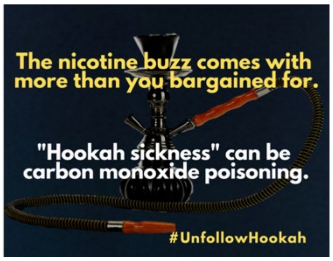

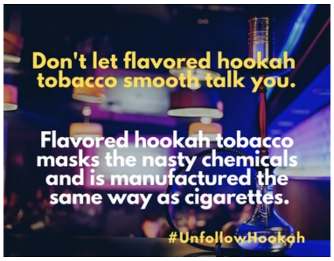

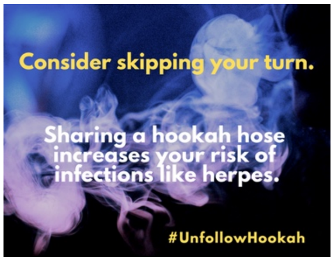

Addiction

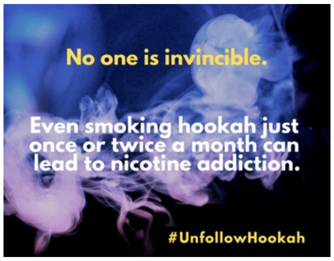

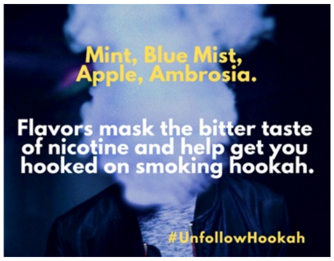

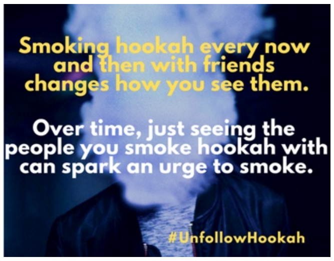

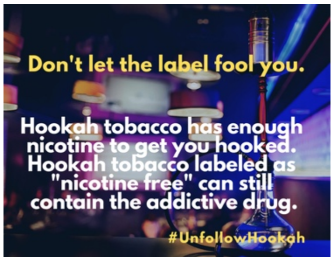

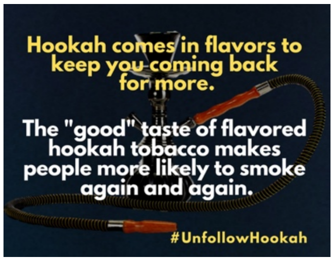

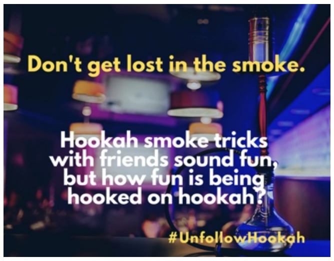

Supplement: Supplementary file 1 [file ijerph-17-08752-s001.pdf]
